# Implementation and early effects of medicaid policy interventions to promote racial equity in pregnancy and early childhood outcomes in Pennsylvania: protocol for a mixed methods study

**DOI:** 10.1186/s12913-024-10982-5

**Published:** 2024-04-22

**Authors:** Marian Jarlenski, Evan Cole, Christine McClure, Sarah Sanders, Marquita Smalls, Dara D Méndez

**Affiliations:** 1 Department of Health Policy and Management, University of Pittsburgh School of Public Health, 130 DeSoto St, A619, 15261 Pittsburgh, PA USA; 2 Department of Epidemiology, University of Pittsburgh School of Public Health, Pittsburgh, PA USA

**Keywords:** Medicaid, Pregnancy, Well-child visits, Prenatal care, Health policy, Health equity, Mixed methods

## Abstract

**Background:**

There are large racial inequities in pregnancy and early childhood health within state Medicaid programs in the United States. To date, few Medicaid policy interventions have explicitly focused on improving health in Black populations. Pennsylvania Medicaid has adopted two policy interventions to incentivize racial health equity in managed care (equity payment program) and obstetric service delivery (equity focused obstetric bundle). Our research team will conduct a mixed-methods study to investigate the implementation and early effects of these two policy interventions on pregnancy and infant health equity.

**Methods:**

Qualitative interviews will be conducted with Medicaid managed care administrators and obstetric and pediatric providers, and focus groups will be conducted among Medicaid beneficiaries. Quantitative data on healthcare utilization, healthcare quality, and health outcomes among pregnant and parenting people will be extracted from administrative Medicaid healthcare data. Primary outcomes are stakeholder perspectives on policy intervention implementation (qualitative) and timely prenatal care, pregnancy and birth outcomes, and well-child visits (quantitative). Template analysis methods will be applied to qualitative data. Quantitative analyses will use an interrupted time series design to examine changes over time in outcomes among Black people, relative to people of other races, before and after adoption of the Pennsylvania Medicaid equity-focused policy interventions.

**Discussion:**

Findings from this study are expected to advance knowledge about how Medicaid programs can best implement policy interventions to promote racial equity in pregnancy and early childhood health.

## Background

Rates of maternal and infant morbidity and mortality in the United States far exceed those of comparable nations [1]. The burdens of racist policies have produced vastly worse outcomes for Black and Native, relative to White, populations [2]. For example, Black and Native birthing people are more than three times as likely to experience pregnancy-related mortality compared to white birthing people [3]. For every pregnancy-related death, there are thousands of birthing people who experience severe morbidity; including stark racial disparities where Black populations are more likely to experience stroke or serious cardiovascular events sending them on a trajectory of adverse health outcomes beyond pregnancy [4, 5]. We also see similar racial inequities for infant mortality and morbidity. These racial inequities are not adequately explained by individual behaviors or other socio-economic factors, but are a complex intersection of factors shaped by structural and social determinants [2, 6], policies and institutions carrying out such policies [7]. There is a long history of structural racism that has resulted in practices and policies that have had a detrimental impact on Black and Indigenous populations in the United States [8].

State Medicaid programs are the largest single payer for pregnancy and birth in the US, covering 68% of births to Black people [9]. As such, Medicaid programs have great potential to implement structural interventions to advance racial equity in healthcare and health outcomes during pregnancy and postpartum [10]. Historically, Medicaid policies have promoted equality, that is, providing equal benefits to all regardless of the distribution of need [11]. An equity-focused policy approach, however, will direct resources toward improving health and well-being among those with the greatest need [12]. Unfortunately, a vast body of research conducted among Medicaid-enrolled populations shows that healthcare systems do not provide the same quality of obstetric care to Black people and other people of color, relative to white people [13–18].

Pennsylvania’s Medicaid program is the fourth-largest in the United States, with 3.5 million people enrolled and expenditures of $35.1 billion [19, 20]. Past research in the Pennsylvania Medicaid program has demonstrated Black people were less able to access prenatal and postpartum care relative to those in other race groups [15]. Reporting from the Pennsylvania Maternal Mortality Commission shows that in more than half of the cases of pregnancy-associated deaths, the decadents were enrolled in Medicaid [21]. Similar to national figures, pregnancy-associated death was far more common among Black people vs. those of other races ( [21].

To ameliorate these racial disparities, Pennsylvania Medicaid is currently implementing two novel policies with the goal to advance racial equity in pregnancy and child health. The first, the equity incentive payment program, was initiated in 2020. The equity incentive payment program makes available approximately $26 million in Medicaid managed care organization (MCO) payments each year to plans that improve access to timely prenatal care and well-child visits among Black beneficiaries. The second is the maternity care bundled payment model, initiated in 2021, designed to provide incentives to obstetric providers across a wide range of pregnancy health outcomes and specifically incentivizes improvements among Black beneficiaries.

Although these policy approaches are unique, it is possible that other state Medicaid programs or other health insurers could learn from the policies and adapt or expand these approaches. Our research team will conduct a mixed-methods study to investigate the implementation and early effects of the two aforementioned policy changes on pregnancy and infant health equity. Our research aims are to: (1) evaluate implementation and early effects of the equity incentive payment program prenatal and early childhood healthcare outcomes and experiences among Black Medicaid beneficiaries; and (2) determine the extent to which an equity-focused maternity care bundled payment model affects racial equity in obstetric care and pregnancy health outcomes. To achieve these aims, we will draw on established partnerships between university researchers, community organizations, and policymakers to collect and analyze data. First, we will collect qualitative data with diverse stakeholders including Medicaid beneficiaries, MCO plan representatives, and pediatric and obstetric care clinicians to study implementation of these equity-focused policy changes. Second, we will use a community-partnered approach to develop a quantitative analysis plan of Medicaid administrative data for an estimated 167,000 birthing person-child dyads to estimate early effects of these policies. Our cross-disciplinary, community-engaged partnerships will enable us to triangulate how the healthcare policy structures of state Medicaid programs can be changed to promote racial equity in health.

## Methods

### Theoretical framework

The proposed research seeks to advance knowledge about the causes of, and structural interventions to improve, health and well-being among Black pregnant and parenting persons and their children in Medicaid. The theoretical model underlying this work is informed by foundational literature from a range of disciplines. First, it incorporates Critical Race Theory and Public Health Critical Race Praxis, which posit structural determinants, such as racism and other forms of oppression (e.g., sexism, classism, poverty), as fundamental causes of adverse social environments that interact to make certain populations more susceptible to illness and resulting in suboptimal health [22–26]. Second, it incorporates political science theory that dominant social definitions of populations shape group empowerment and resulting health policies and material benefits [27]. Third, it draws on new scholarship suggesting the necessity of studying social welfare policies with a critical race lens centering the beneficiaries’ lived experiences [11, 28, 29].

**Fig. 1:**
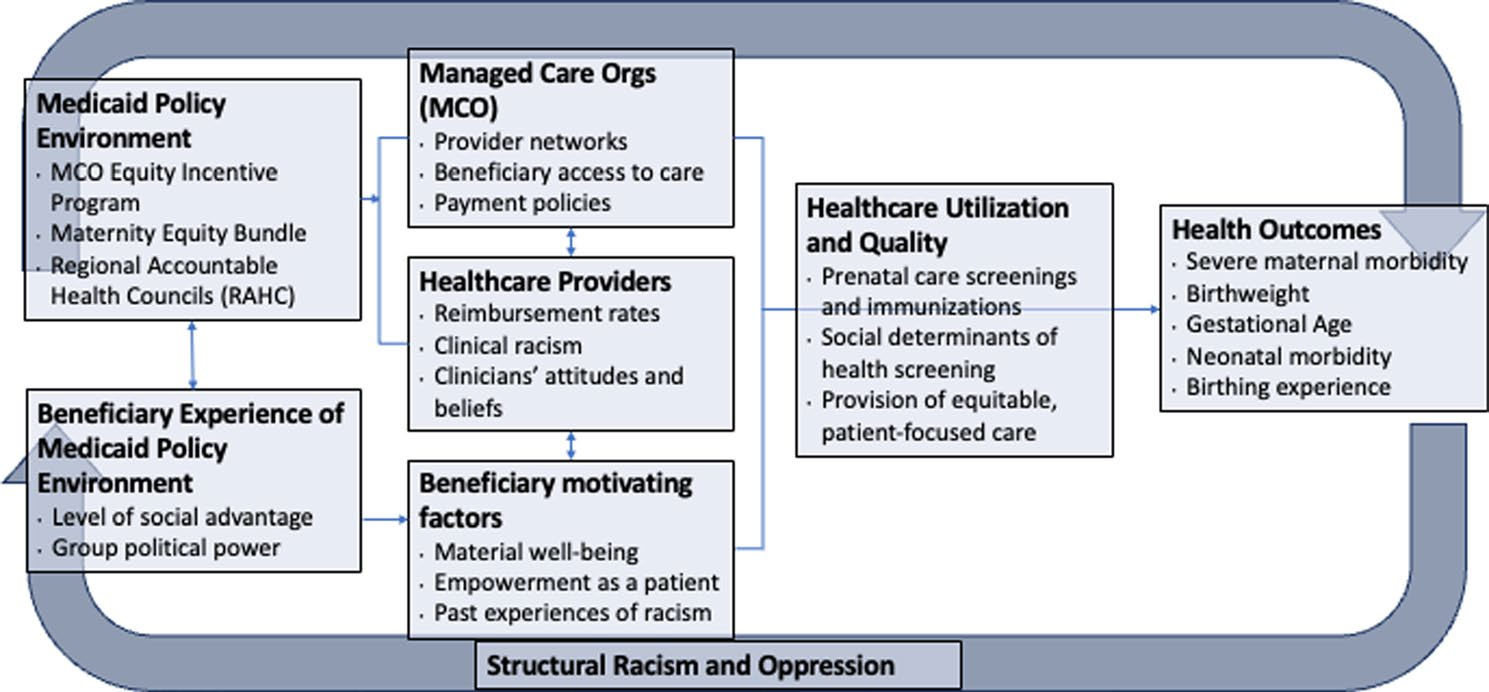
Conceptual model of the evaluation of structural interventions in Medicaid to promote racial equity in pregnancy and child health

As depicted in Fig. 1, our research project identifies both the Medicaid policy environment as well as the beneficiary experiences of the policy environment as upstream factors that influence healthcare organization and beneficiaries’ interaction with healthcare systems. In particular, we aim to facilitate and further enhance the connection between beneficiaries’ lived experiences and policy decision-makers through our collaboration with community partners. This connection can influence the policymaking process that shapes how care is delivered both at the managed care and healthcare provider levels. Healthcare utilization and quality are conceptualized as intermediate outcomes which may influence pregnancy and birth outcomes.

### Medicaid policy interventions

**Fig. 2:**
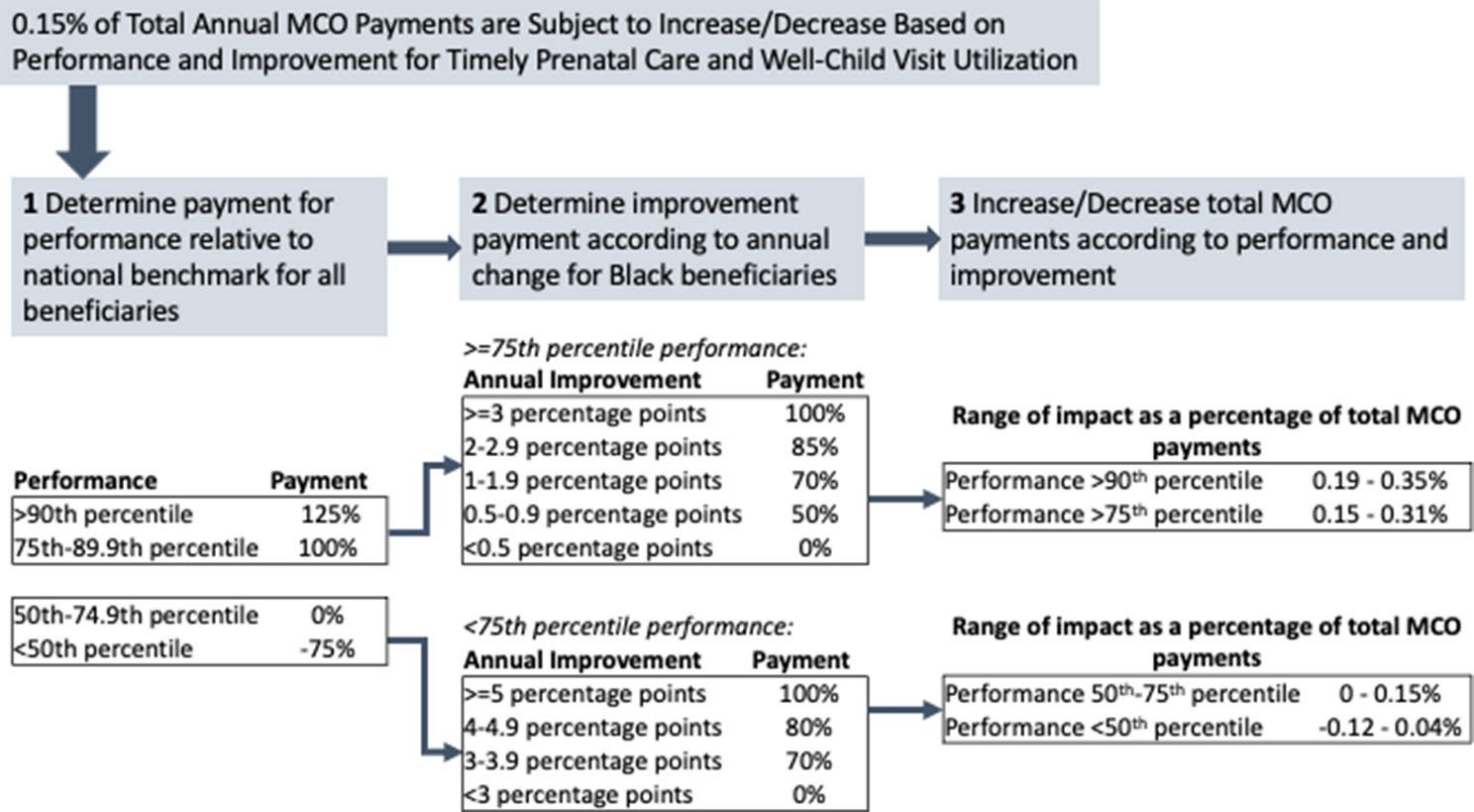
Pennsylvania Medicaid’s health equity incentive payment program will condition payments to managed care organizations based on overall performance as well as improvement among Black beneficiaries

Nearly all Medicaid beneficiaries in Pennsylvania are enrolled in 1 of 8 Medicaid managed care plans, which manage the physical health care of enrollees and are subject to pay-for-performance requirements for healthcare quality measures. Currently, the Pennsylvania Medicaid program makes available 2% of total payments to MCO plans, contingent on MCO plan performance on 13 different healthcare quality metrics. An equity incentive payment program was added to this reimbursement scheme for two metrics in 2020: timely prenatal care and well-child visit utilization in the first 15 months of life (Fig. 2). Specifically, 2/13 (or 0.15%) of total payments are withheld for these two equity-focused metrics. MCO plans are assessed on overall performance and subsequently on the annual improvement on these measures among Black beneficiaries. MCO plans can be penalized (up to -0.12% of total payments) or rewarded (up to + 0.35% of total payments) for their performance on each of these two metrics.

**Fig. 3:**
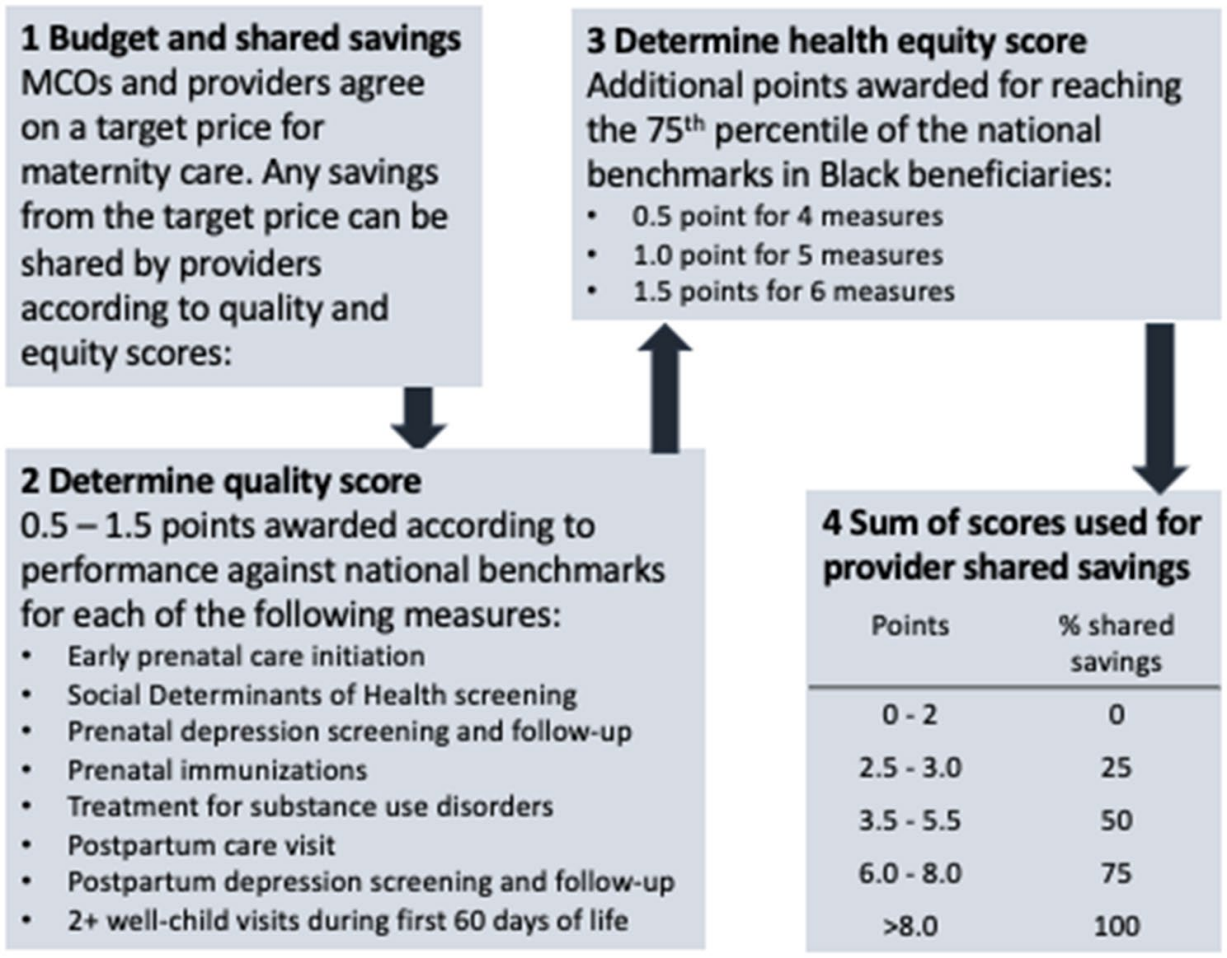
Pennsylvania Medicaid’s equity-focused maternity bundled payment model will allow for shared savings between obstetric care providers and managed care organizations, allowing for extra shared savings among providers whose Black patients experience better outcomes

Second, Pennsylvania Medicaid implemented a maternity care bundled payment model in 2021 that considers outcomes among Black beneficiaries (Fig. 3). Under maternity care bundled payment models, obstetric providers are incentivized to meet a total cost threshold and quality metrics for prenatal and delivery care [30]. Specifically, providers and payers agree on a target cost for low- or average-risk perinatal care, including pregnancy, delivery, and postpartum care. If total payments to providers are lower than the target cost while maintaining certain quality metrics, providers and payers share those savings. Under Pennsylvania’s new model, providers are able to achieve shared savings based on quality metric performance, as well as a health equity score reflecting performance on those metrics among Black beneficiaries.

### Qualitative data Collection

To understand the interventions and responses to these policies, as well as related implementation barriers and facilitators, we will conduct interviews with each at least two representatives from each MCO (*n* = 18). We will partner with colleagues from the Department of Human Services (DHS) to identify relevant MCO representatives. Interviews will elucidate MCOs’ perspectives, processes used by MCOs to design their interventions (e.g., review of existing evidence, input from community members or providers who serve them), anticipated effects, and sustainability of these payment policy changes. The goal is for some of the results of these interviews to inform our understanding of the implementation process which will be further explored in the interviews and focus groups with clinicians and Medicaid recipients.

In collaboration with the Community Health Advocates (CHA) program led by Healthy Start Pittsburgh, as well as other community and organizational partners across the state, we will recruit current and former Medicaid beneficiaries for focus group participation. We aim to recruit ∼ 50 community participants and will purposively oversample Black participants and will aim to recruit people of all ethnicities who identify as Black and multi-racial in order to achieve our aims of elucidating the experiences of Black parenting and pregnant people in Medicaid. Inclusion criteria are: current pregnancy or pregnant within the past 2 years; current or former enrollment in Pennsylvania Medicaid and/or Healthy Start; and ability to complete the interview in English.

Finally, we will partner with colleagues from DHS to identify pediatric and obstetric health professionals for interviews regarding the maternity bundled payment program and key outcomes related to the equity incentive payment. We will also use Medicaid administrative data to identify providers who serve Black beneficiaries and invite them to participate. We will aim to interview at least 20 obstetric and pediatric healthcare professionals to elucidate their perspectives on how structural racism in medicine affects patient outcomes, and the types of Medicaid policy changes that should be implemented.

We developed separate focus group/interview guides for community members, MCO leaders, and healthcare professionals. Each interview guide consists of open-ended questions to elucidate participants’ experiences with Medicaid; desired policy changes in Medicaid (among beneficiary participants); perceived steps that would be useful to combat anti-Black racism in healthcare and social services (especially among Black participants); and perspectives about the new Medicaid policies. Additionally, the interview guides ask demographic questions regarding gender identity, race, and ethnicity. We will first pilot-test the guide with our research partners and Healthy Start CHAs for clarity of question wording. All interviews will take place in-person in a private office space, or over the phone or videoconference, according to participants’ preferences and COVID-19 protocols. The interviewer will describe study objectives to each participant, obtain consent, and each interview will be audio-recorded and the interviewer will take notes throughout. Interview audio recordings will be transcribed verbatim, and transcripts spot-checked against the audio recordings for accuracy. The audio recording files will then be deleted to protect confidentiality of participants.

### Qualitative data analysis

Study data will be analyzed and reported using the Consolidated Criteria for Reporting Qualitative Research (COREQ) Framework [31]. To analyze data, we will use template analysis, which combines features of deductive content analysis and inductive grounded theory, thereby allowing us to obtain specific information while also capturing any new or unanticipated themes [32]. Two coders will separately code the first 3 interview transcripts, meet to compare codes, discuss inconsistency in coding approaches, and then alter or add codes. This iterative process will be repeated until the coding scheme is fully developed. The coders will independently code all transcripts, and any coding discrepancies will be resolved via discussion. Once coding is complete, synthesis of content will begin by organizing codes under broader domains (meta-codes) as well as sub-codes. The primary analysis will be to convey qualitative data, including the use of illustrative quotes.

### Quantitative healthcare data and analysis

Administrative healthcare data from the Pennsylvania Medicaid program, with linked birthing person-child dyads, will be used to create our quantitative analytic data. Medicaid data include a census of enrollment, hospital, outpatient/professional, pharmaceutical, and provider data for all beneficiaries in the Pennsylvania Medicaid program. Importantly, data contain self-reported race and ethnicity that is provided at the time of Medicaid enrollment (< 2% missing); as well as time-varying data on 9-digit ZIP code of residence. Data also include the amounts paid from Medicaid MCOs to healthcare providers for all medical services. Table 1 shows baseline data from Pennsylvania Medicaid-enrolled persons with a livebirth delivery in 2019, overall and stratified by race of the birthing person. We will also conduct sensitivity analyses to examine dyads that are multi-racial.

**Table 1:** Characteristics of Pennsylvania Medicaid beneficiaries with a livebirth delivery in 2019

	Overall, *n* = 57,270	Non-hispanic black, *n* = 14,622	Non-hispanic white, *n* = 26,508
Age [yrs; mean (SD)]	27.6 (5.7)	27.5 (5.7)	27.6 (5.6)
**Race and Ethnicity** ^**a**^ **, %**			
Non-Hispanic White	46.3	--	100
Non-Hispanic Black	25.5	100	--
Hispanic/Latina/x	19.7	--	--
Others	3.4	--	--
Unknown	5.2	--	--
**Urban residence** ^**b**^ **, %**	88.2	98.0	77.5
**Baseline Outcomes**			
Timely prenatal care, %	86.8	86.0	91.2
**Severe maternal morbidity** ^**c**^ **, %**	3.6	4.2	3.5
Preterm birth (< 37 weeks’ gestation), %	14.0	17.0	13.2

a Race and ethnicity reported by beneficiaries at the time of Medicaid enrollment

b According to the Rural-Urban Commuting Area codes from the US Dept of Agriculture

c According to the Centers for Disease Control and Prevention algorithm

We will employ a comparative interrupted time series (ITS) analyses with a nonequivalent comparison group to estimate policy effects. Specifically, we will evaluate: (1) the extent to which the equity incentive payment program improved timely prenatal care and well-child visits among Black beneficiaries, relative to those of other races; and (2) the extent to which healthcare provider participation in the equity-focused maternity bundled payment model improved healthcare and health outcomes among Black beneficiaries, relative to those of other races.

For Aim 1, outcomes include binary measures of initiating prenatal care in the first trimester, and children receiving at least 6 well-child visits in the first six months of life. We will compare outcomes among Black beneficiaries relative to those of other racial groups, post- relative to pre- implementation of the equity incentive payment program. For Aim 2, outcomes include a composite of prenatal care quality measures (social determinants of health screening, prenatal and postpartum depression screening and follow-up, immunization, screening and treatment for substance use disorders, postpartum visit attendance), gestational age and birthweight, and severe maternal morbidity [33]. For both aims, multivariable regression models will control for maternal age, ethnicity, parity, ZIP code of residence, MCO plan enrollment, Medicaid eligibility category (expansion, pregnancy, disability, or others), and indices of obstetric and pediatric clinical comorbidities [34, 35].

### Sensitivity analyses

Analyses are designed to estimate *early effects* of the policies and should be interpreted alongside the qualitative results regarding policy implementation and beneficiary experiences. One assumption of ITS analyses is that our comparison groups approximate a counterfactual scenario for the intervention groups [36–38]. Although trends in Black-White inequities in pregnancy and child outcomes have, unfortunately, persisted over time [39], the COVID-19 pandemic has differentially burdened Black and Latina/x people relative to other race and ethnic groups [40, 41]. Effects of the pandemic on pregnancy outcomes are only just beginning of what is to be explored [42]. It is therefore possible that we will not be able to disentangle policy effects from effects of COVID-19. To address this limitation, we will employ area-level rates of COVID-19 infection as control variables and for Aim 1 (equity incentive payment) we will conduct a sub-analysis investigating trends in 2021 vs. 2020. We chose to evaluate outcomes for Aim 2 (maternity care bundled payment) only in 2021, comparing the statistical intervention of race*provider. Finally, our qualitative analyses will provide context on differential impacts of COVID-19, which will inform interpretation of the quantitative results.

This study was approved by the University of Pittsburgh Institutional Review Board (Study # 23090108).

## Discussion

This mixed-methods research will investigate the extent to which changes in the Pennsylvania Medicaid program are associated with improvements in access to medical care and health outcomes among Black pregnant and birthing persons and their children. Our past research found that Black childbearing people in Pennsylvania Medicaid consistently experienced worse healthcare and health outcomes, compared to those of other racial and ethnic groups [43, 44]. Racism in healthcare and other systems manifests in systematically worse access to and quality of care and other services for Black childbearing people [8]. In addition to suboptimal healthcare experiences, historical policies and practices such as residential redlining and segregation have resulted in lower wealth attainment in Black communities resulting in inequities in neighborhood factors and perinatal health [45–47].

The policies under study involve modifying common Medicaid reimbursement arrangements– namely, pay-for-performance programs and maternity care bundled payments. The policies are modified to embed financial incentives for Medicaid health plans and healthcare providers to improve the quality of care and health outcomes for Black pregnant and parenting persons and their children. These are the first such payment policies, to our knowledge, that explicitly aim to promote racial health equity with an explicit focus on addressing inequities that affect Black and Indigenous populations in Pennsylvania.

Interest from policymakers in payment reforms to improve health equity has increased recently; however, information on the implementation and effects of such models is sparse [48, 49]. Although several state Medicaid programs have adopted maternity care bundled payment models, prior models have not considered racial inequities in pregnancy outcomes [30, 50]. In 2012, Oregon adopted regional health equity coalitions as part of the state Medicaid program’s transition to Coordinated Care Organizations (CCOs). CCOs were required and given support to develop strategies that would address racial health disparities within the Medicaid population, and the regional health equity coalitions included underrepresented stakeholders to guide CCOs in the development of these interventions. While CCOs did reduce Black-white differences in primary care utilization and access to care within 3 years of policy implementation, it did not impact disparities in emergency department utilization [51]. The current research project will add to the extant evidence on how Medicaid programs can use policy to promote racial health equity.

Our study is limited in investigating the direct effects of the pandemic on racial inequities in perinatal and infant health and the intersections between the effects of the pandemic and the effects of the aforementioned Medicaid policies. However, we will have the ability to look at changes in outcomes over time. Additionally, these payment reform interventions focus largely on transforming the financing and delivery of healthcare, drawing attention to the structural and social determinants of health in the healthcare system. It is estimated that medical care contributes 10–20% to health outcomes; health and well-being are also shaped by factors such as environmental and socioeconomic conditions [52].

This study will contribute to the current body of knowledge about the recent interventions in Medicaid focused on racial equity. Specifically, findings will shed light on how the equity-focused obstetric care policies are being implemented and provide an evaluation of effects on health outcomes. These results can be used for future adaptions of the policy interventions or to inform the adoption of such equity-focused policies in different geographic regions of the United States.

## Data Availability

No datasets were generated or analysed during the current protocol study.

## Abbreviations

MCO: Managed Care Organization
CHA: Community Health Advocate
CCO: Coordinated Care Organization
